# Vitamin D Status among Young Children Aged 1–3 Years: A Cross-Sectional Study in Wuxi, China

**DOI:** 10.1371/journal.pone.0141595

**Published:** 2015-10-27

**Authors:** Xin Zhao, Jianping Xiao, Xiangpeng Liao, Liyi Cai, Fei Xu, Daozhen Chen, Jingying Xiang, Rui Fang

**Affiliations:** 1 Department of Obstetrics, Wuxi Maternity and Child Health Hospital Affiliated to Nanjing Medical University, Wuxi, Jiangsu, China; 2 Centre for Reproductive Medicine, Wuxi Maternity and Child Health Hospital Affiliated to Nanjing Medical University, Wuxi, Jiangsu, China; 3 Department of Child healthcare and Newborn, Wuxi Maternity and Child Health Hospital Affiliated to Nanjing Medical University, Wuxi, Jiangsu, China; 4 Central Laboratory, Wuxi Maternity and Child Health Hospital Affiliated to Nanjing Medical University, Wuxi, Jiangsu, China; Nanjing Medical University, CHINA

## Abstract

**Background:**

The increasingly recognized importance of vitamin D has been discussed and vitamin D status among young children has attracted widespread attention in recent years. However, study on vitamin D status in young children aged 1–3 y is limited in China.

**Objective:**

To evaluate the nutritional vitamin D status of young children aged 1–3 y in Wuxi, southeastern China.

**Methods:**

A large cohort of 5,571 young children aged 1–3 y were recruited in this study who visited the child health clinics at the Wuxi Maternity and Child Health Hospital (latitude 31.57°N) during January 2014 to January 2015. Wuxi was located in southeastern China at a latitude of 31.57°N. Finger-stick blood sampling was conducted in all the subjects and serum 25-Hydroxyvitamin D (25(OH)D) levels were measured to evaluate their vitamin D status.

**Results:**

In this study, serum 25(OH)D levels of young children at the age of 1–3 years ranged from 20.6–132.9 nmol/L (Median: 71.5 nmol/L). 16.1% of the population had vitamin D deficiency (<50 nmol/L), while 38.8% of the subjects had a sufficient (50–74.9 nmol/L) vitamin D level. An optimal vitamin D status (≥75 nmol/L) was found in 45.1% of the young children. The prevalence of vitamin D deficiency was higher in autumn (19.5%) than in summer (12.1%). There was no significant difference in vitamin D status between genders. The binary logistic regression analysis revealed that child age was strongly associated with vitamin D deficiency (adjusted OR: 1.173; 95%CI: 1.053–1.308; P = 0.004).

**Conclusions:**

The prevalence of vitamin D deficiency was 16.1% among young children aged 1–3 y in Wuxi. Season and child age were associated with their vitamin D status. It is implied that young children should receive adequate amounts of vitamin D supplementation and spend more time outdoors to prolong the sunlight exposure when they grow older.

## Introduction

Vitamin D deficiency is common in the general population around the world [1]. More than one billion people were estimated to have an insufficient vitamin D status. As is known, only 5%-10% of vitamin D throughout the body is obtained from dietary intake, while more than 90% of vitamin D derives from cutaneous production [2]. Under the effect of ultraviolet-B irradiation, vitamin D precursor 7-dehydrocholesterol in the skin is converted into pre-vitamin D_3_, which then spontaneously isomerizes to vitamin D_3_ (cholecalciferol). Vitamin D_3_ is transported to the liver where it is converted into 25(OH)D [3]. Finally in the kidney, 25(OH)D is catalyzed by 1α-hydroxylase to transform into the active form, 1,25(OH)_2_D [4]. Vitamin D plays an important role in various physiological processes. Long-term of vitamin D deficiency, in addition to its well-known skeletal effects [5], not only influences the calcium and phosphorous absorption, but also increases risks of various chronic diseases, such as autoimmune diseases, asthma, cardiovascular diseases and infections [6–10]. It has been reported that the lack of vitamin D is associated with metabolic syndromes in children [11–12]. The recent finding claims that vitamin D deficiency is also in relation to depression indicating that vitamin D might be involved in regulating development of the neural system [13–14].

Rapid growth in childhood makes them in great demand of nutrients including vitamin D. Nevertheless, many studies have shown that the children population is at high risks of vitamin D deficiency [15–16]. A proper diet can not satisfy the daily vitamin D requirement as the amount of vitamin D in the natural foods is limited. Children spending less time on outdoor activities are likely to have a lower serum vitamin D level [17]. A number of studies have suggested that infants should receive a reasonable amount of vitamin D supplements after birth [18–19]. However, an agreement on the optimal serum vitamin D concentration for well-being has not been reached worldwide [20–21], and data relevant to vitamin D status of Chinese children from large population studies is scarce which leads to the lack of consensus and established guidelines for vitamin D supplementation in Chinese toddlers.

25(OH)D is relatively stable with a long half-life period in serum, thus it is widely used as a marker to evaluate the nutritional status of vitamin D [22]. Our previous study on maternal vitamin D status during the second trimester of pregnancy in Wuxi has found a high prevalence (78.7%) of vitamin D deficiency (<50 nmol/L) [23]. Several studies have indicated a strong positive correlation between neonatal vitamin D status and maternal vitamin D during pregnancy [24–25]. Young children aged 1–3 y have obvious changes in diets and lifestyles compared with neonates. Our study aims to evaluate the vitamin D status by measuring serum 25(OH)D concentration in young children aged 1–3 y from a large population in Wuxi. Wuxi city is located in the south of Jiangsu province, a developed region in southeastern China with a subtropical monsoon climate, where the Han Chinese population has similar dietary habits. The Han Chinese constitute a majority (>99.5%) of the population in the south of Jiangsu. Our study will provide information supporting vitamin D supplementation guidelines for toddlers in Jiangsu province and in the other regions of southeastern China.

## Materials and Methods

### Ethics statement

This study was a hospital-based cross-sectional study. Participants were recruited from the population of young children aged 1–3 y during their regular follow-up visit to Child health clinics of Wuxi Maternal and Child Health Hospital from January 2014 to January 2015. The guardians of these young children were informed about this research and its objectives, and written consent was obtained from them. This study was approved by the Medical Research Ethics Board of Wuxi Maternity and Child Health Hospital.

### Sample collection and 25(OH)D assay

200μL of blood sample was collected by using a finger stick for each participant and placed directly into a 0.5mL microtube. Within 10 min after collection, specimens were centrifuged at 3500 rpm for 15 min. Serum samples were stored at -80°C until assay. Serum 25(OH)D concentrations of the participants were measured by using enzyme-linked immunosorbent assay following the manufacturer’s instructions (IDS Ltd., Boldon Colliery, Tyne & Wear, UK). The inter-assay and intra-assay coefficients of variation were <10%.

### Statistical analysis

Vitamin D status was categorized as deficient [25(OH)D level <50 nmol/L], sufficient [25(OH)D level 50–74.9 nmol/L] and optimal [25(OH)D level ≥75 nmol/L] according to the Medical Research guidelines described previously [26–27]. The season for serum sample collection was classified as follows: spring (from March to May), summer (from June to August), autumn (from September to November), and winter (from December to February). Data was presented using median and 25th-75th percentile (p25-p75) values. All the statistical analysis was performed using the Statistical Package for the Social Sciences statistical software package version 20.0 (SPSS Inc, Chicago, Illinois, USA).The difference among continuous variables was assessed by Mann–Whitney U-test or Kruskal–Wallis test, and Chi Square test was used to assess the significance among categorical variables [23]. The difference was considered as statistically significant when P<0.05. The binary logistic regression model which included age and season associated with vitamin D in the univariate analysis (P<0.05) were used to determine the association between age and vitamin D deficiency after adjusting for confounders.

## Results

**Fig 1:**
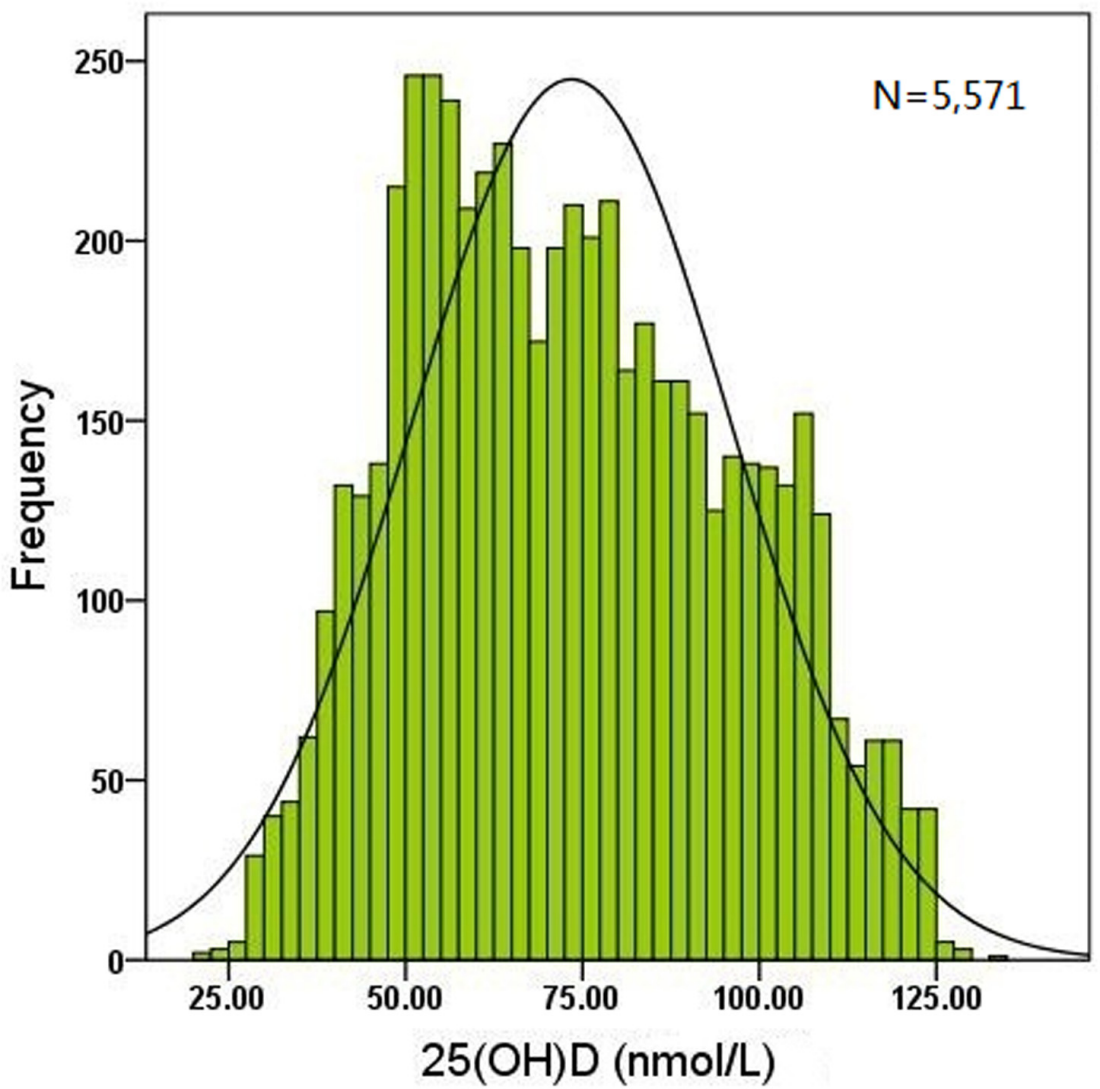
Frequency distribution of the serum 25(OH)D concentration in 5,571 young children. The serum 25(OH)D level ranged from 20.6–132.9 nmol/L with a median value of 71.5 nmol/L and most values of 25(OH)D levels were more than 50 nmol/L.

**Fig 2:**
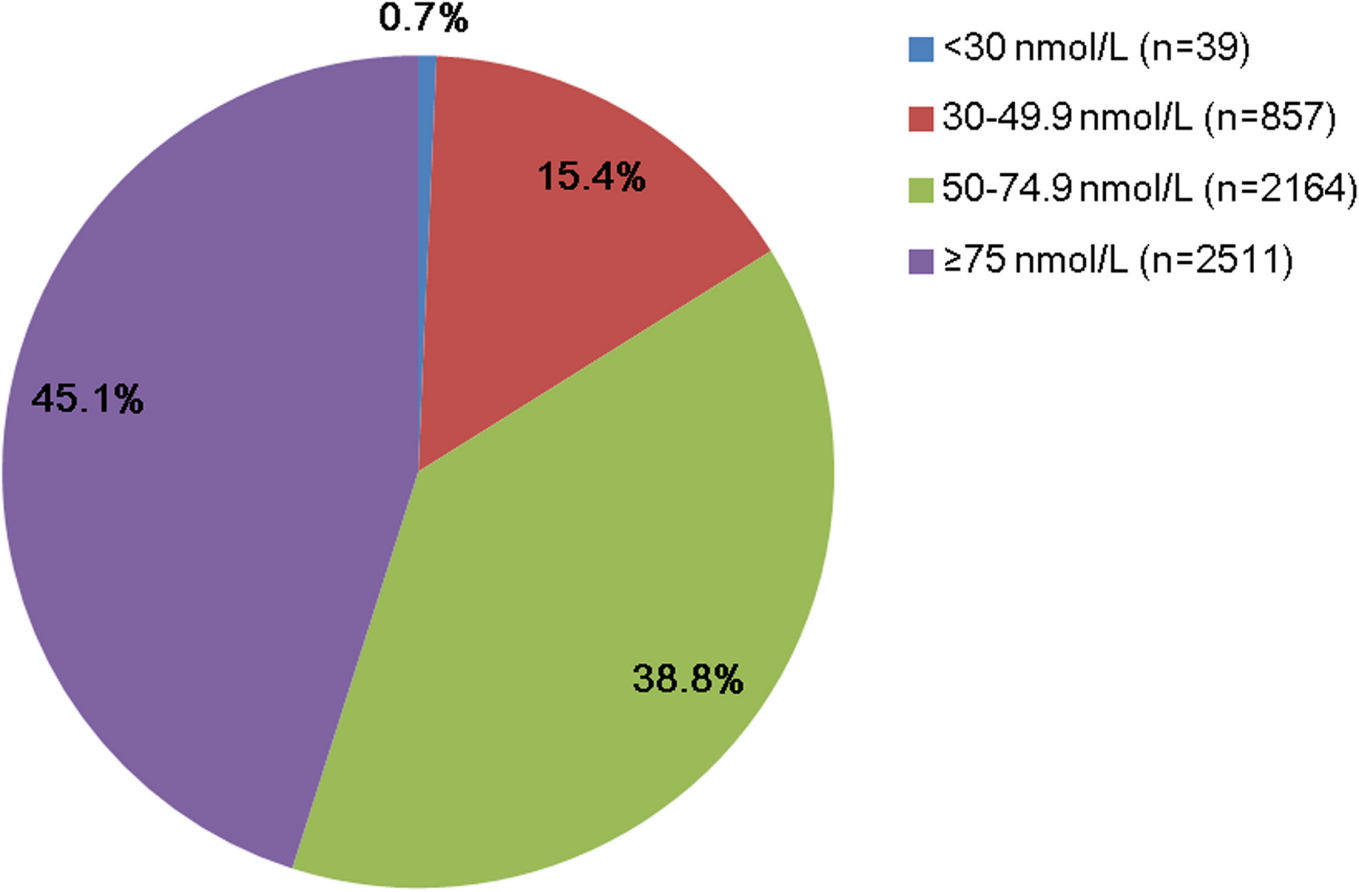
Proportion of different categories of the serum 25(OH)D level in the study population. Serum 25(OH)D levels were sectioned into four categories indicated by different color (<30 nmol/L, 30–49.9 nmol/L, 50–74.9 nmol/L and ≥75 nmol/L). n indicated children number in each category.

A population of 5,571 young children (2,903 boys and 2,668 girls) aged 1–3 y was recruited in this study. The frequency distribution of serum 25(OH)D concentration showed that most values were > 50 nmol/L. The serum 25(OH)D level ranged from 20.6–132.9 nmol/L with a median value of 71.5 nmol/L (Fig 1). In the whole cohort, 16.1% of the subjects were found to be deficient of vitamin D (<50 nmol/L), while 38.8% had a sufficient (50–74.9 nmol/L) vitamin D status. An optimal 25(OH)D concentration (≥75 nmol/L) was found in 45.1% of the population (Fig 2).

**Fig 3:**
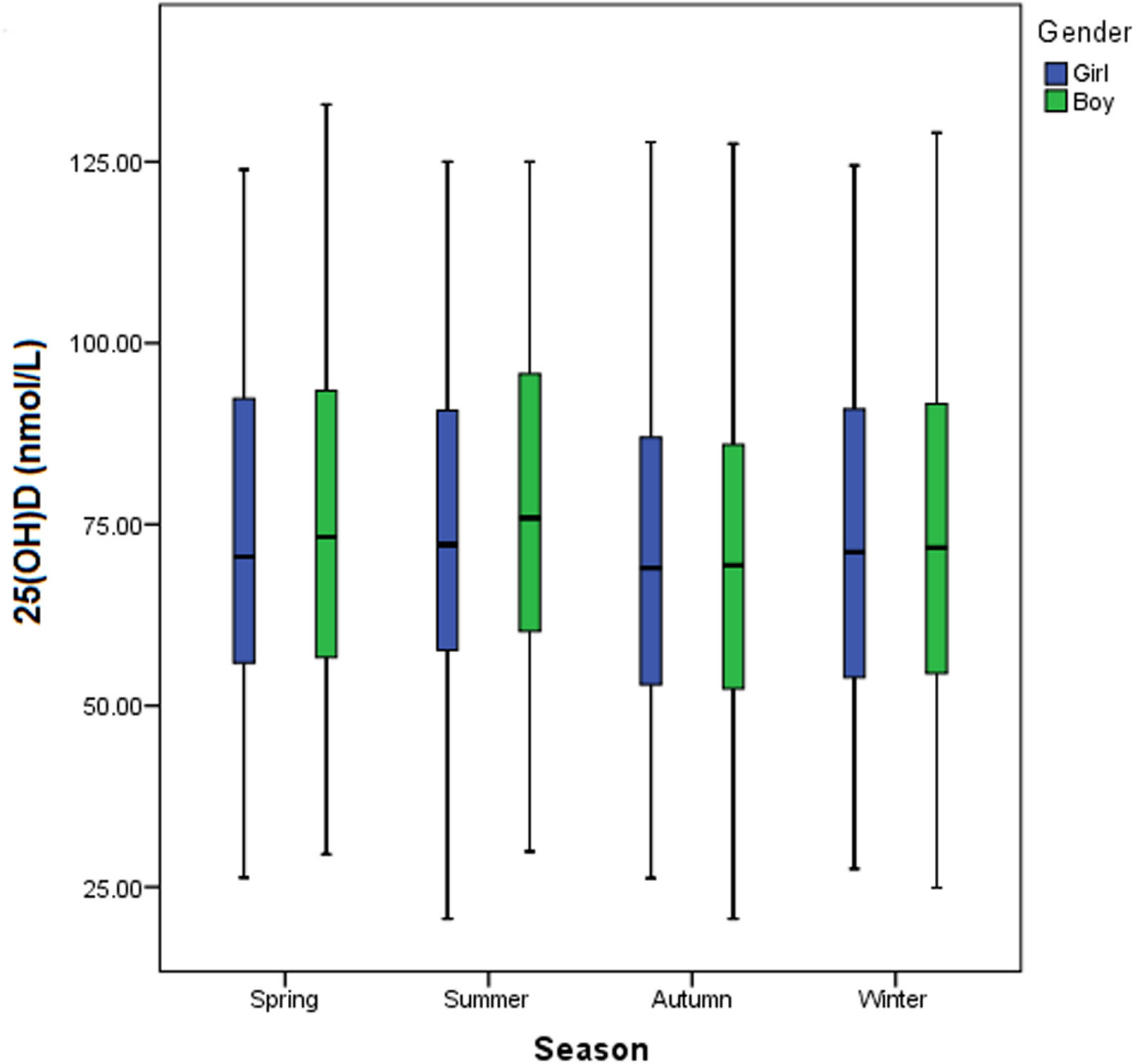
Comparison of the serum 25(OH)D concentration among young children in different seasons. The serum 25(OH)D level was highest in summer and lowest in autumn.

**Fig 4:**
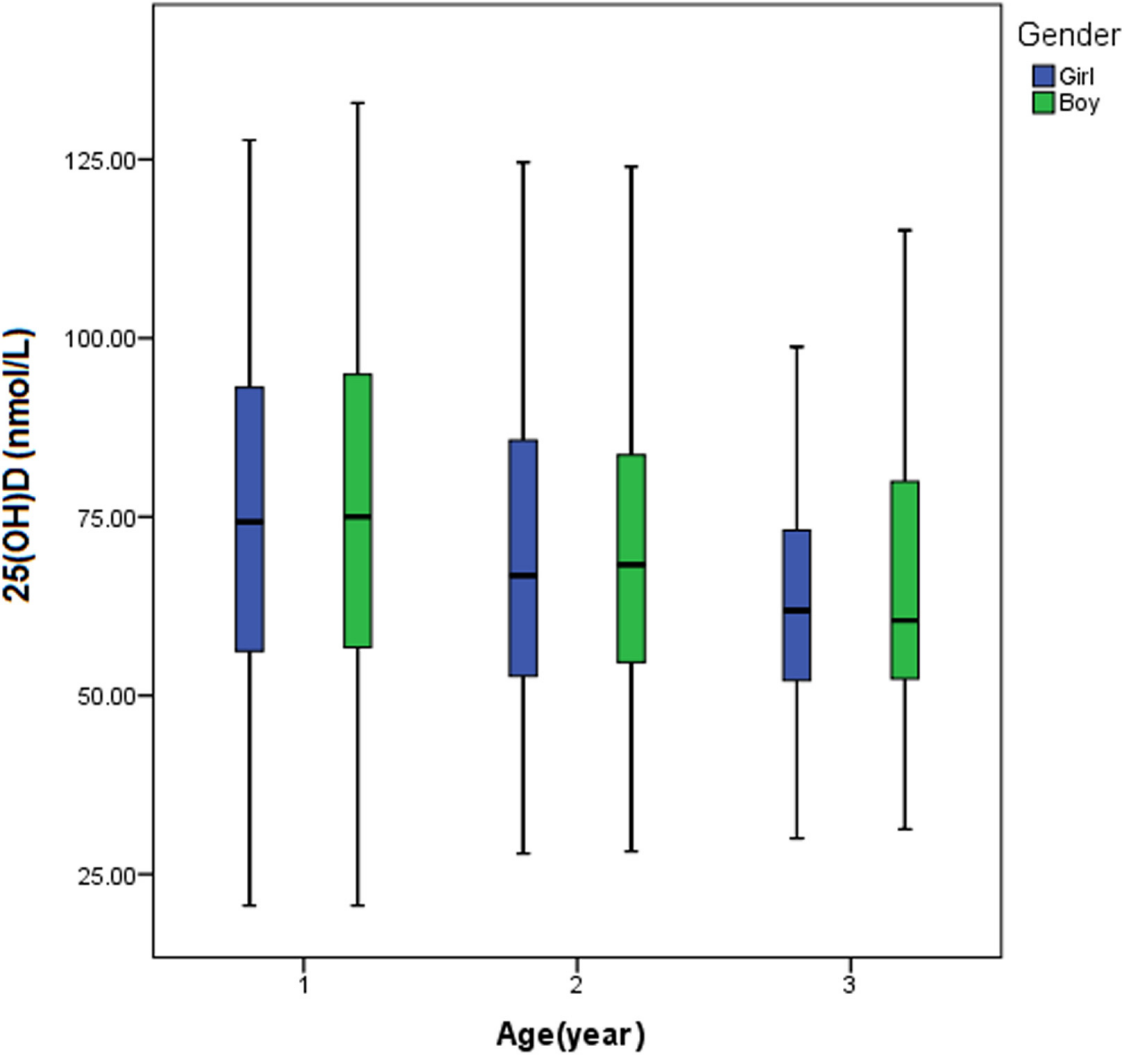
Comparison of the serum 25(OH)D concentration among young children at different ages. As the children grew older, the median serum 25(OH)D level was decreased.

The median 25(OH)D concentration in boys (n = 2,903) and in girls (n = 2,668) was 72.3 and 70.8 nmol/L, respectively. However, they did not differ significantly (P = 0.121) (Table 1). The serum 25(OH)D level varied remarkably with the change in seasons, which was highest in summer (median: 74.4 nmol/L) and lowest in autumn (median: 69.0 nmol/L) (Table 1, Fig 3). When the comparison was made between every two seasons, significant differences in the serum 25(OH)D level were found between spring and autumn (P<0.001), summer and winter (P = 0.001), summer and autumn (P<0.001), and between autumn and winter (P = 0.003). However, the difference in the serum concentration of 25(OH)D was not significant between spring and winter (P = 0.098) or between spring and summer (P = 0.076). Remarkable variation of the serum 25(OH)D level was found in different age groups (P<0.001) (Table 1). As the children grew older, the median serum 25(OH)D level was decreased (Fig 4).

**Table 1:** Comparison of serum 25(OH)D levels in 5,571 young children stratified by age, season or gender.

Group	N	Serum 25(OH)D (nmol/L)	
		Median	Percentile (p25-p75)
**Age**			
1 y ^a^ ^,^ ^b^	3845	74.70	56.40–94.25
2 y ^a^ ^,^ ^c^	1266	67.60	53.48–84.93
3 y ^b^ ^,^ ^c^	460	61.00	52.30–76.75
**Season**			
Spring ^d^ ^,^ ^e^ ^,^ ^f^	1256	71.6	56.3–93.3
Summer ^d^ ^,^ ^g^ ^,^ ^h^	907	74.4	58.7–93.3
Autumn ^e^ ^,^ ^g^ ^,^ ^i^	1471	69.0	52.6–86.6
Winter ^f^ ^,^ ^h^ ^,^ ^i^	1937	71.4	54.3–91.2
**Gender**			
Boy ^j^	2903	72.3	55.5–91.2
Girl ^j^	2668	70.8	54.7–90.3

Values of serum 25(OH)D levels were compared using Mann–Whitney U test.

^a^ significant difference between children aged 1y and aged 2y (P<0.001).

^b^ Significant difference between children aged 1y and aged 3y (P<0.001).

^c^ Significant difference between children aged 2y and aged 3y (P<0.001).

^d^ No significant difference between spring and summer (P = 0.076).

^e^ Significant difference between spring and autumn (P<0.001).

^f^ No significant difference between spring and winter (P = 0.098).

^g^ Significant difference between summer and autumn (P<0.001).

^h^ Significant difference between summer and winter (P = 0.001).

^i^ Significant difference between autumn and winter (P = 0.003).

^j^ No significant difference between boys and girls (P = 0.121).

There was also a significant difference in the vitamin D status of young children among various season and age groups (P<0.001) but not between genders (P = 0.149) (Table 2). The prevalence of optimal vitamin D (≥75 nmol/L) was highest in summer (49.1%) and lowest in autumn (41.4%), which was 45.9% in spring and 45.5% in winter, respectively. Similarly, the prevalence of vitamin D deficiency (<50 nmol/L) also changed greatly in different seasons which was lowest in summer (12.1%) and highest in autumn (19.5%). Child age was also an important determinant of nutritional vitamin D status. As the children grew older, the prevalence of optimal vitamin D (≥75 nmol/L) was decreased which was 49.6%, 37.9% and 27.2% in children aged 1, 2 and 3, respectively. Meanwhile, the prevalence of vitamin D deficiency (<50 nmol/L) was increased which was 15.2%, 17.8% and 18.7% in children aged 1, 2 and 3, respectively.

**Table 2:** Categories of vitamin D status by age, season or gender (n = 5,571).

		Vitamin D Status				
Group	N^a^	<30nmol/L	30–49.9nmol/L	50–74.9nmol/L	≥75nmol/L	
		n (%)^b^	n (%)^b^	n (%)^b^	n (%)^b^	P value^c^
**Age**						<0.001
1 y	3845	34 (0.9%)	551 (14.3%)	1354 (35.2%)	1906 (49.6%)	
2 y	1266	5 (0.4%)	220 (17.4%)	561 (44.3%)	480 (37.9%)	
3 y	460	0 (0%)	86 (18.7%)	249 (54.1%)	125 (27.2%)	
**Season**						<0.001
Spring	1256	2 (0.2%)	159 (12.7%)	519 (41.3%)	576 (45.9%)	
Summer	907	4 (0.4%)	106 (11.7%)	352 (38.8%)	445 (49.1%)	
Autumn	1471	17 (1.2%)	269 (18.3%)	576 (39.1%)	609 (41.4%)	
Winter	1937	16 (0.8%)	323 (16.7%)	717 (37.0%)	881 (45.5%)	
**Gender**						0.149
Boy	2903	23 (0.8%)	419 (14.4%)	1127 (38.8%)	1334 (46.0%)	
Girl	2668	16 (0.6%)	438 (16.4%)	1037 (38.9%)	1177 (44.1%)	

^a^ N, the total number of children in each group.

^b^ Within each group, Vitamin D status was presented as the number (percent) of young children in different categories.

^c^ Values were compared using the Chi Square (χ^2^) test. P<0.001 indicated that the difference in the prevalence of diverse vitamin D status among different age or season groups was significant.

**Fig 5:**
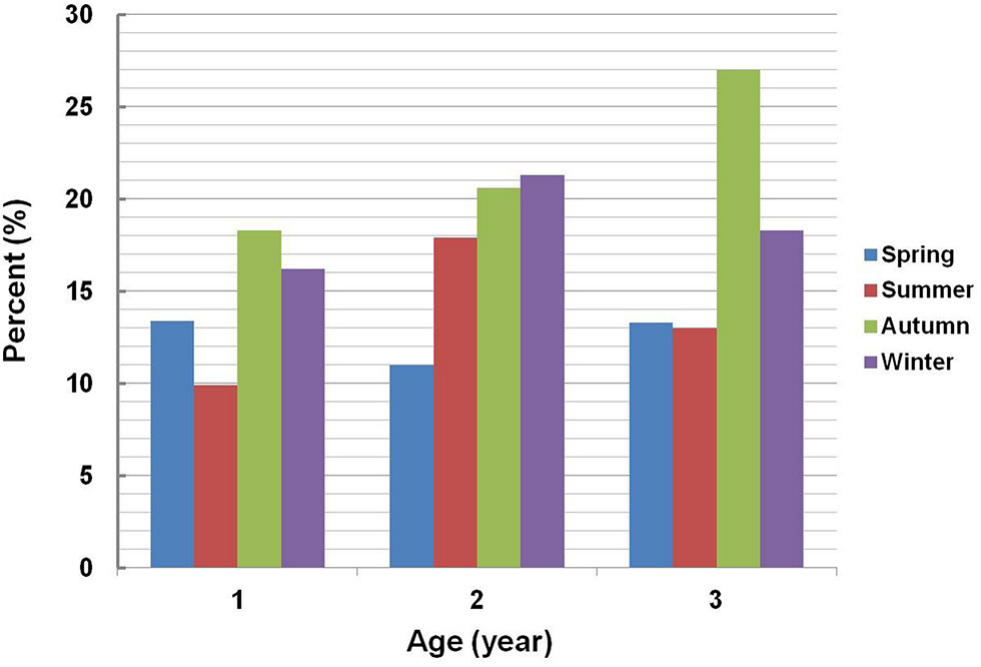
Comparison of the prevalence of vitamin D deficiency in young children by age and season. Vitamin D deficiency was defined as a serum 25(OH)D level of <50 nmol/L. All the children were stratified by age into three subgroups (aged 1, 2 and 3). In each subgroup, the subjects were further stratified by season of serum collection. The prevalence of vitamin D deficiency in each season was calculated separately within each age group.

Seasonal variation of the prevalence of vitamin D deficiency (<50 nmol/L) was found to be diverse among children of different ages (Fig 5). For the group of children aged 1 or 3, the prevalence of vitamin D deficiency was highest in autumn (18.3% or 27.0%) and lowest in summer (9.9% or 13.0%), respectively, while it was highest in winter (21.3%) and lowest in spring (11.0%) for children aged 2.

It was indicated by binary logistic regression analysis that season (P<0.001) and age (P = 0.009) were associated with the risk of vitamin D deficiency in children aged 1–3. After controlling for seasons, the risk of vitamin D deficiency was increased as children grew older (adjusted OR: 1.173; 95%CI: 1.053–1.308; P = 0.004).

## Discussion

Our study investigated the nutritional vitamin D status of young children aged 1–3 y from a large population in Wuxi. The prevalence of vitamin D deficiency (<50 nmol/L) was 16.1%, and 54.9% of the toddlers had a serum 25(OH)D level less than 75 nmol/L. Geographic characteristics and household socioeconomic background are important determinants of vitamin D status [17]. The distribution of vitamin D status varies in different regions according to the duration of sunlight exposure. Dietary intakes of vitamin D are influenced by dietary habits in the population of different regions. Wuxi (31.57°N) is located within an economic developed region in the southeastern China and children here might have relatively high intakes of fish, milk and dairy products rich in vitamin D. An investigation conducted in Hangzhou (30.3°N), a city also located in southeastern China approximately 210 kilometers from Wuxi, showed that the prevalence of serum 25(OH)D <50 nmol/L was 21.9% among children aged 2–5 y (n = 1221) [15]. A large population-based study of young children aged 1–3 y (n = 1979) in Guangzhou (23.1°N), southern China, reported that 19.2% of the subjects had a serum 25(OH)D level of <50 nmol/L [28]. Both of the two studies presented a similar prevalence of vitamin D deficiency (<50 nmol/L) as ours. In view of the potential difference in dietary habits and sunshine duration, our result might at least represent the vitamin D status for the young children population aged 1–3 y in the developed regions in southeastern China.

Another survey on vitamin D status among infants aged 6–23 months in Alaska USA, where more than 50% of the general population lives at a latitude >58°N, found that 11% and 20% of the subjects had a 25(OH)D level of less than 32.5 nmol/L and of 32.5–62.5 nmol/L, respectively [29]. The other investigation in rural Nepal (27.39°N) reported that vitamin D status in 91.1% of the young children aged 12–60 months was deficient [30]. All these studies suggest that the nutritional vitamin D status in young children of different countries varies markedly. Genetic variants related to vitamin D synthesis and bioavailability by ethnics might account for the vitamin D level diversity [31–32]. Investigations on the vitamin D status of Chinese children from different ethnic groups will be indispensible and urgent as China is a multi-ethnic country [33].

We did not observe any significant difference in serum 25(OH)D concentration between boys and girls among our population. In line with many studies on vitamin D status of pre-school children and school-age children, our study also found that the vitamin D level of young children was decreased and the risk of vitamin D deficiency was increased while they grew older [6,7,16,34]. It implies that long-term assessment of vitamin D status and vitamin D supplementation should be an important part of the clinical follow-up and healthconscious behaviors for young children. Generally speaking, the diet of Chinese children particularly at the age of 1–3 y changes gradually from breast milk and infant formula fortified with vitamin D supplementation to natural foods, while their lifestyles such as sedentary behavior also changes and they would like to spend more time on outdoor activities. In addition to seasons and racial profiles, diet and ultraviolet-B skin exposure are important environmental factors correlated with nutritional vitamin D status in young children of different ages.

Several strengths of this study include that it is a cross-sectional study with a large population representative of young children aged 1–3 y in Wuxi. Our study also has potential limitations and thus should be considered with caution. In this study, anthropometric measurements and sociodemographic characteristics of the participants were not collected. This hospital-based cross-sectional study prevents us to rule out bias and to obtain detailed information for their lifestyle factors and dietary structure which might have a key effect on nutritional vitamin D status in young children. To the best of our knowledge, this study represents the largest sample size investigation of vitamin D status in Chinese toddlers.

In conclusion, this study evaluated the vitamin D status of 5,571 young children aged 1–3 y living in Wuxi, southeastern China and assessed its association with gender, season and age. We found a low prevalence of vitamin D deficiency in this population. Season and child age other than gender was associated with vitamin D levels. While the children grew older, the risk of vitamin D deficiency was increased implying a potential imbalance between nutritional intakes and requirements. Therefore it is suggested that long-term follow-up and vitamin D assessment should be a routine practice in pediatric clinics as a prophylactic means for vitamin D deficiency in young children.

## References

[1] Calvo MS, Whiting SJ, Barton CN (2005) Vitamin D intake: a global perspective of current status. J Nutr 135: 310–316. 15671233 10.1093/jn/135.2.310

[2] Holick MF (2008) Sunlight, UV-radiation, vitamin D and skin cancer: how much sunlight do we need? Adv Exp Med Biol 624: 1–15. 10.1007/978-0-387-77574-6_1 18348443

[3] Holick MF, MacLaughlin JA, Clark MB, Holick SA, Potts JT Jr, Anderson RR, et al (1980) Photosynthesis of previtamin D3 in human skin and the physiologic consequences. Science 210: 203–205. 6251551 10.1126/science.6251551

[4] Ponsonby AL, Lucas RM, Lewis S, Halliday J (2010) Vitamin D status during pregnancy and aspects of offspring health. Nutrients 2: 389–407. 10.3390/nu2030389 22254029 PMC3257641

[5] Zerofsky M, Ryder M, Bhatia S, Stephensen CB, King J, Fung EB (2015) Effects of early vitamin D deficiency rickets on bone and dental health, growth and immunity. Matern Child Nutr 2015 4 7. 10.1111/mcn.12187 PMC4610869 25850574

[6] Holmlund-Suila E, Koskivirta P, Metso T, Andersson S, Makitie O, Viljakainen HT (2013) Vitamin D deficiency in children with a chronic illness-seasonal and age-related variations in serum 25-hydroxy Vitamin D concentrations. PLoS One 8: e60856 10.1371/journal.pone.0060856 23585857 PMC3621868

[7] Gilbert-Diamond D, Baylin A, Mora-Plazas M, Marin C, Arsenault JE, Hughes MD, et al (2010) Vitamin D deficiency and anthropometric indicators of adiposity in school-age children: a prospective study. Am J Clin Nutr 92: 1446–1451. 10.3945/ajcn.2010.29746 20926524 PMC3131841

[8] Zhang HQ, Teng JH, Li Y, Li XX, He YH, He X, et al (2014) Vitamin D status and its association with adiposity and oxidative stress in schoolchildren. Nutrition 30: 1040–1044. 10.1016/j.nut.2014.02.024 25102819

[9] Litonjua AA (2012) Vitamin D deficiency as a risk factor for childhood allergic disease and asthma. Curr Opin Allergy Clin Immunol 12: 179–185. 10.1097/ACI.0b013e3283507927 22266772 PMC3315849

[10] Thornton KA, Marin C, Mora-Plazas M, Villamor E (2013) Vitamin D deficiency associated with increased incidence of gastrointestinal and ear infections in school-age children. Pediatr Infect Dis J 32: 585–593. 10.1097/INF.0b013e3182868989 23340562

[11] Buyukinan M, Ozen S, Kokkun S, Saz EU (2012) The relation of vitamin D deficiency with puberty and insulin resistance in obese children and adolescents. J Pediatr Endocrinol Metab 25: 83–87. 22570955 10.1515/jpem-2011-0426

[12] Mellati AA, Sharifi F, Faghihzade S, Mousaviviri SA, Chiti H, Kazemi SA (2015) Vitamin D status and its associations with components of metabolic syndrome in healthy children. J Pediatr Endocrinol Metab 28: 641–648. 10.1515/jpem-2013-0495 25928755

[13] Almeida OP, Hankey GJ, Yeap BB, Golledge J, Flicker L (2015) Vitamin D concentration and its association with past, current and future depression in older men: The Health In Men Study. Maturitas 81: 36–41. 10.1016/j.maturitas.2015.01.016 25724592

[14] Gracious BL, Finucane TL, Friedman-Campbell M, Messing S, Parkhurst MN (2012) Vitamin D deficiency and psychotic features in mentally ill adolescents: a cross-sectional study. BMC Psychiatry 12: 38 10.1186/1471-244X-12-38 22571731 PMC3441857

[15] Zhu Z, Zhan J, Shao J, Chen W, Chen L, Li W, et al (2012) High prevalence of vitamin D deficiency among children aged 1 month to 16 years in Hangzhou, China. BMC Public Health 12: 126 10.1186/1471-2458-12-126 22330045 PMC3312872

[16] Neyestani TR, Hajifaraji M, Omidvar N, Eshraghian MR, Shariatzadeh N, Kalayi A, et al (2012) High prevalence of vitamin D deficiency in school-age children in Tehran, 2008: a red alert. Public Health Nutr 15: 324–330. 10.1017/S1368980011000188 21356149

[17] Voortman T, van den Hooven EH, Heijboer AC, Hofman A, Jaddoe VW, Franco OH (2015) Vitamin D deficiency in school-age children is associated with sociodemographic and lifestyle factors. J Nutr 145: 791–798. 10.3945/jn.114.208280 25833782

[18] Hatun S, Ozkan B, Orbak Z, Doneray H, Cizmecioglu F, Toprak D, et al (2005) Vitamin D deficiency in early infancy. J Nutr 135: 279–282. 15671226 10.1093/jn/135.2.279

[19] Wilne S, Collier J, Kennedy C, Koller K, Grundy R, Walker D (2007) Presentation of childhood CNS tumours: a systematic review and meta-analysis. Lancet Oncol 8: 685–695. 17644483 10.1016/S1470-2045(07)70207-3

[20] Hollis BW (2005) Circulating 25-hydroxyvitamin D levels indicative of vitamin D sufficiency: implications for establishing a new effective dietary intake recommendation for vitamin D. J Nutr 135: 317–322. 15671234 10.1093/jn/135.2.317

[21] Greer FR (2003) Vitamin D deficiency—it's more than rickets. J Pediatr 143: 422–423. 14571210 10.1067/S0022-3476(03)00465-7

[22] Lips P (2007) Relative value of 25(OH)D and 1,25(OH)2D measurements. J Bone Miner Res 22: 1668–1671. 17645404 10.1359/jbmr.070716

[23] Xiao JP, Zang J, Pei JJ, Xu F, Zhu Y, Liao XP (2015) Low maternal vitamin D status during the second trimester of pregnancy: a cross-sectional study in Wuxi, China. PLoS One 10: e0117748 10.1371/journal.pone.0117748 25659105 PMC4320063

[24] Monangi N, Slaughter JL, Dawodu A, Smith C, Akinbi HT (2014) Vitamin D status of early preterm infants and the effects of vitamin D intake during hospital stay. Arch Dis Child Fetal Neonatal Ed 99: F166–168. 10.1136/archdischild-2013-303999 23852093 PMC3933171

[25] Song SJ, Si S, Liu J, Chen X, Zhou L, Jia G, et al (2013) Vitamin D status in Chinese pregnant women and their newborns in Beijing and their relationships to birth size. Public Health Nutr 16: 687–692. 10.1017/S1368980012003084 23174124 PMC10271381

[26] Holick MF, Binkley NC, Bischoff-Ferrari HA, Gordon CM, Hanley DA, Heaney RP, et al (2011) Evaluation, treatment, and prevention of vitamin D deficiency: an Endocrine Society clinical practice guideline. J Clin Endocrinol Metab 96: 1911–1930. 10.1210/jc.2011-0385 21646368

[27] Palacios C, Gonzalez L (2014) Is vitamin D deficiency a major global public health problem? J Steroid Biochem Mol Biol 144 Pt A: 138–145. 24239505 10.1016/j.jsbmb.2013.11.003 PMC4018438

[28] Liu Xiaomin, Zhu Bing, Guo Min, Lin Tao, Chen Yi, Zhao Mingqi, et al (2014) 25-hydroxyvitamin D status in 11,524 children aged 0–14 in Guangzhou. Int J Lab Med 35: 1226–1227. (In Chinese)

[29] Gessner BD, Plotnik J, Muth PT (2003) 25-hydroxyvitamin D levels among healthy children in Alaska. J Pediatr 143: 434–437. 14571215 10.1067/S0022-3476(03)00410-4

[30] Avagyan D, Neupane SP, Gundersen TE, Madar AA (2015) Vitamin D status in pre-school children in rural Nepal. Public Health Nutr Apr 1: 1–7. 10.1017/S136898001500083X PMC10271105 25827017

[31] Suaini NH, Koplin JJ, Ellis JA, Peters RL, Ponsonby AL, Dharmage SC, et al (2014) Environmental and genetic determinants of vitamin D insufficiency in 12-month-old infants. J Steroid Biochem Mol Biol 144 Pt B: 445–454. 25174667 10.1016/j.jsbmb.2014.08.018

[32] Kuhn T, Kaaks R, Teucher B, Hirche F, Dierkes J, Weikert C, et al (2014) Dietary, lifestyle, and genetic determinants of vitamin D status: a cross-sectional analysis from the European Prospective Investigation into Cancer and Nutrition (EPIC)-Germany study. Eur J Nutr 53: 731–741. 10.1007/s00394-013-0577-8 24005870

[33] Arguelles LM, Langman CB, Ariza AJ, Ali FN, Dilley K, Price H, et al (2009) Heritability and environmental factors affecting vitamin D status in rural Chinese adolescent twins. J Clin Endocrinol Metab 94: 3273–3281. 10.1210/jc.2008-1532 19549746 PMC2741721

[34] Nichols EK, Khatib IM, Aburto NJ, Serdula MK, Scanlon KS, Wirth JP, et al (2015) Vitamin D status and associated factors of deficiency among Jordanian children of preschool age. Eur J Clin Nutr 69: 90–95. 10.1038/ejcn.2014.142 25117992 PMC7607367

